# The Effect of Bacteriophage Preparations on Intracellular Killing of Bacteria by Phagocytes

**DOI:** 10.1155/2015/482863

**Published:** 2015-12-09

**Authors:** Ewa Jończyk-Matysiak, Marzanna Łusiak-Szelachowska, Marlena Kłak, Barbara Bubak, Ryszard Międzybrodzki, Beata Weber-Dąbrowska, Maciej Żaczek, Wojciech Fortuna, Paweł Rogóż, Sławomir Letkiewicz, Krzysztof Szufnarowski, Andrzej Górski

**Affiliations:** ^1^Bacteriophage Laboratory, Ludwik Hirszfeld Institute of Immunology and Experimental Therapy, Polish Academy of Sciences, Weigla 12, 53-114 Wroclaw, Poland; ^2^Phage Therapy Unit, Medical Center of the Ludwik Hirszfeld Institute of Immunology and Experimental Therapy, Polish Academy of Sciences, Weigla 12, 53-114 Wroclaw, Poland; ^3^Department of Clinical Immunology, Transplantation Institute, Medical University of Warsaw, Nowogrodzka 59, 02-006 Warsaw, Poland

## Abstract

Intracellular killing of bacteria is one of the fundamental mechanisms against invading pathogens. Impaired intracellular killing of bacteria by phagocytes may be the reason of chronic infections and may be caused by antibiotics or substances that can be produced by some bacteria. Therefore, it was of great practical importance to examine whether phage preparations may influence the process of phagocyte intracellular killing of bacteria. It may be important especially in the case of patients qualified for experimental phage therapy (approximately half of the patients with chronic bacterial infections have their immunity impaired). Our analysis included 51 patients with chronic Gram-negative and Gram-positive bacterial infections treated with phage preparations at the Phage Therapy Unit in Wroclaw. The aim of the study was to investigate the effect of experimental phage therapy on intracellular killing of bacteria by patients' peripheral blood monocytes and polymorphonuclear neutrophils. We observed that phage therapy does not reduce patients' phagocytes' ability to kill bacteria, and it does not affect the activity of phagocytes in patients with initially reduced ability to kill bacteria intracellularly. Our results suggest that experimental phage therapy has no significant adverse effects on the bactericidal properties of phagocytes, which confirms the safety of the therapy.

## 1. Introduction

Intracellular killing of bacteria (IKB) is one of the fundamental defense mechanisms against invading pathogens. Phagocytic cells (neutrophils, monocytes, tissue macrophages, and dendritic cells) are a component of innate immunity [1]. They are involved in nonspecific defense of the body against external pathogens and substances produced by them, for example, toxins, which consists of antigen uptake and formation of a phagosome and then the destruction of the antigens there, due to the presence of degrading enzymes [2, 3]. High effectiveness in killing bacteria is achieved in neutrophils by combining the action of reactive oxygen species (ROS) and substances contained in the granules of these cells (proteins that mediate the antibacterial activity of phagocytes in anaerobic conditions) [4].

Innate immune deficiencies, antibiotic therapy, and the development of strategies allowing bacteria to survive inside the phagocyte are possible causes of defects in the IKB, which is one of the key steps of phagocytosis [5]. Bacterial infections, including those caused by antibiotic-resistant bacterial strains, are a major cause of morbidity in patients with immune deficits, such as transplant recipients, cancer patients, and persons with primary immunodeficiency or secondary immunodeficiency, for example, AIDS. Individuals with defects in phagocyte function often suffer from severe recurrent infections [6–8] due to impaired immunity. The spectrum of microorganisms causing infections in these patients extends beyond the microorganisms that cause infections in people with a properly functioning immune system. This leads to infection with microorganisms, which in healthy individuals do not cause infection. In patients with immunodeficiencies, an increased probability of disease incidence caused by bacteria resistant to antibiotics was observed. Due to the fact that certain antibiotics impair phagocytic bactericidal functions, their usage may cause weakened immunity of the patient. The use of antibiotics can lead to a decrease in the capacity of phagocytes for phagocytosis and IKB [9–11]. As was shown by Méhes et al., 2012, the phagocytosis and IKB are impaired in the case of granulocytes (isolated from healthy donors) killing* S. aureus* strain resistant to vancomycin (heterogeneous vancomycin-intermediate* Staphylococcus aureus*, hVISA) and methicillin (methicillin-resistant* Staphylococcus aureus*, MRSA) [12].* S. aureus* isolates, insensitive to antibiotics, showed greater resistance to opsonophagocytosis and IKB than methicillin-sensitive isolates (methicillin-sensitive* Staphylococcus aureus*, MSSA). The hVISA strains are characterized by a thickened cell wall, with a modified bond in the peptidoglycan. This suggests that structural changes which result in loss of sensitivity to antibiotics also cause bacterial resistance to opsonophagocytosis and killing by phagocytes.

More knowledge of the immunogenicity of phages and their possible interactions with the immune system cells is necessary for the rational application of phages in patients. Previous studies have shown that the use of phage preparations is safe [13–15]. It was shown that the T4 phage and its proteins: the major capsid protein (gp23), head vertex protein (gp24), highly immunogenic outer capsid protein (Hoc), and small outer capsid protein (Soc) on the surface of the head, do not stimulate cytokine production, notably proinflammatory cytokines such as IL-1*α*, IL-6, IL-12, and TNF-*α* [15]. Also, the effect of phages on the production of reactive oxygen species (ROS), which are produced by phagocytes after the uptake of an antigen, was investigated [13, 15, 16]. ROS are a strong “weapon” used by phagocytic cells to eliminate pathogens that are engulfed in the process of phagocytosis. Previous* in vitro* findings suggest that the use of phage preparations for treatment of systemic infections could reduce the harmful effects of bacterial cell lysis products on patients' tissues and organs (e.g., during sepsis the production of ROS may result in tissue and organ damage).

In view of the presented data it was of great practical importance to examine whether phage preparations may influence the process of phagocyte IKB. This phenomenon may be important especially in the case of patients qualified for EPT, as, according to our phage therapy center, approximately half of the patients with chronic bacterial infections have their immunity impaired [14, 17]. Therefore, it was reasonable to examine this phenomenon in patients with chronic infections who had previously been treated with antibiotics and in whom this treatment had not given a positive effect. We hope that this research will help answer the question of whether applying phage preparations may cause further alterations in IKB.

## 2. Materials and Methods

### 2.1. Ethics Statement

Blood samples were taken from patients qualified for experimental phage therapy (EPT) in the Phage Therapy Unit in Wroclaw under the protocol of “experimental phage therapy of drug-resistant bacterial infections, including MRSA infections” [14] and were collected from each patient before and during or after EPT. The study was approved by the Bioethics Committee at the Wroclaw Medical University (approval numbers: KB-349/2005, KB-236/2010, KB-448/2010, KB-461/2012, KB-722/2012, and KB-81/2013) and was conducted in accordance with the Declaration of Helsinki. Data was anonymized and deidentified prior to analysis. In experiments peripheral blood phagocytes, polymorphonuclear neutrophils (PMNs) and mononuclear blood cells (PBMCs), from healthy donors were used based on the approval of the Bioethics Committee (approval number: KB519/2009).

### 2.2. Patients

Heparinized blood samples were taken from patients suffering from chronic bacterial infections in whom prior antibiotic treatment had not brought an improvement (e.g., eradication of bacteria) at different stages of the EPT: before the therapy, during phage treatment (between the 5th day and the 168th day of the therapy), and after the therapy (5–55 days). Mean time of treatment was 50 days. Our analysis included 51 patients with chronic Gram-negative and Gram-positive bacterial infections (including patients with diabetes, *n* = 6). The patients were diagnosed to suffer from chronic urinary tract infection (*n* = 18, including chronic bacterial prostatitis, *n* = 5), ulceration (*n* = 13), fistula (*n* = 12), and respiratory tract infection (*n* = 8). Phage lysates were used by the patients topically (*n* = 27), rectally (*n* = 12), orally (*n* = 5), and in combination: topical and oral route (*n* = 7). Due to the limited number of unique patients, the test groups were not equal in size. The control group consisted of 39 healthy volunteers.

The effectiveness of the treatment was evaluated after the EPT, according to the scale (from A to G; when the result was described as A–C the effect was classified as a good response to the therapy, and D–G represented an inadequate response) described by Międzybrodzki et al. (2012) [14].

### 2.3. Materials

Phage lysates received by the patients came from the collection of the Bacteriophage Laboratory of the IIET PAS or the Institute of Biotechnology, Sera and Vaccines BIOMED SA. Those from BIOMED were the phage preparations used by patients against* S. aureus* (A3/R, fi 200/6409, P4/6409, 676/Ż, and MS-1),* E. faecalis* (1C/K and 15/P), and* P. aeruginosa* (col 21, F-8, 119x, and MP-1). The patients received the preparations in the range of phage titer 10^6^–10^9^ pfu/mL depending on the individual properties of the phage (stability). Patients used phage preparations active against the following bacterial strains:* E. coli*: coli 93/1349, coli 99/DSM, coli 3/2057, coli 104/Lek, and coli 54/181;* E. faecalis*: 1C/K, Ent28/794, Ent38/794, Ent56/1854, Ent17/NW, Ent39/276, and Ent 15/P;* P. aeruginosa*: F-8/LBF, 119x, PsMN/3486, col 21, Ps21, and MP-1;* S. aureus*: 676/Ż, fi200/6409, A3/R, P4/6409, and MS-1.

The pathogens that caused chronic infections were isolated from patients and used to test the bactericidal activity of phagocytes:* Staphylococcus aureus* (*n* = 23),* Pseudomonas aeruginosa* (*n* = 11),* Escherichia coli* (*n* = 7),* Enterococcus faecalis* (*n* = 7),* K. oxytoca* (*n* = 2), and* S. marcescens* (*n* = 1). Bacterial strains isolated from patients that are used for testing the bactericidal activity of phagocytes in healthy donors were the following: Gram-positive:* Staphylococcus aureus* 28500,* Staphylococcus aureus* 28265, and* Enterococcus faecalis* 28197, and Gram-negative:* Pseudomonas aeruginosa* 28420 and* Escherichia coli* 28217.

### 2.4. Preparation of Bacterial Suspensions

Bacterial suspensions for IKB experiments were prepared according to Buisman et al. (1991) [18]. Overnight culture from agar slants was centrifuged (1300 ×g, 4°C, *t* = 15 min) and washed with phosphate-buffered saline (PBS). It was diluted appropriately for every strain, and the optical density of each sample was determined. The bacterial suspension was diluted with PBS to obtain 5 × 10^8^ cells/mL. Then universal human AB serum (Sigma-Aldrich) was added to the bacterial suspension in order to achieve a 10% final concentration in the sample. Samples were incubated at 37°C for 25 min. Then the bacteria were washed in PBS (1300 ×g, 4°C, *t* = 10 min). The precipitate was suspended in PBS.

### 2.5. Isolation of Phagocytes from Peripheral Blood Samples

PBMCs and PMNs were isolated in a density gradient (a double gradient of Histopaque 1119 and Histopaque 1077 (Sigma-Aldrich)) by centrifugation (700 g, 30 min, 20°C) [19]. Then the PMN-rich layer of Histopaque 1119 and mononuclear-cell-rich layer of Histopaque 1077 were collected, and the cells were washed (5 min, 870 ×g, 4°C) in PBS. The percentage of monocytes in the mononuclear cell suspension prepared for the experiment was determined under a fluorescence microscope with the use of Simultest Leucogate (Becton Dickinson) containing a fluorescein-labeled anti-CD45 antibody and anti-CD14 antibody labeled with phycoerythrin. The isolated phagocyte cell fractions were then diluted to achieve the required density: 1 × 10^6^ cells/mL. Cell viability was evaluated.

### 2.6. Study on the Influence of EPT on IKB by Phagocytes

IKB by PBMCs and PMNs isolated from peripheral blood was evaluated according to the method described by Buisman et al. (1991) and Leijh et al. (1982) [18, 20], using in each case a pathogenic bacterial strain isolated from a patient and a standard nonpathogenic* E. coli* B strain. IKB was calculated as the percentage of killed phagocytosed bacteria. Bactericidal activity of peripheral blood phagocytes was tested before, during, and/or after EPT.

PBMCs and PMNs with a density of 1 × 10^6^ cells/mL and opsonized bacteria with a density of 5 × 10^8^ cells/mL were used. In the process of IKB, initial phagocytosis was carried out for 3 min (bacteria opsonized with 10% serum were added into the phagocyte suspension and then phagocytosed). Extracellular bacteria were removed by washing (200 g, 4°C, *t* = 5 min). Cells were suspended in cold PBS. Phagocytes containing bacteria were incubated for 60 min at 37°C (PMNs) and 90 min (PBMCs) in the presence of 10% serum. The IKB process was stopped by adding cold PBS. Then phagocytes containing phagocytosed bacteria were centrifuged (200 g, 4°C, *t* = 5 min), and the precipitate lysed solution of 0.01% albumin (Sigma-Aldrich) was added after 3, 60, and 90 min. The obtained samples were diluted in PBS, dilutions were plated onto agar plates that were incubated for 18 h at 37°C, and the resulting colonies were counted. The IKB by phagocytes (the percentage of killed phagocytosed bacteria) was calculated according to the formula IKB = (1 − *B*/*A*) × 100%, where *A* means the number of all initially phagocytosed bacteria and *B* means the number of phagocytosed bacteria not killed after 60 min incubation with PMNs or after 90 min incubation with PBMCs. Four repetitions of the IKB assay per blood sample were done.

### 2.7. The Effect of Bacteriophage Preparations on the Number of Patients' Peripheral Blood Leukocytes and on the Level of Inflammatory Markers in Blood of Patients Treated with Phage Preparations

Blood smears of patients were made with heparinized fresh venous blood samples taken from patients treated with EPT within 1–3 hours of collection. A drop of blood was applied to the center line of the end of the slide and smeared with a glass slide with the edges polished at an angle of 30–45°. Peripheral blood manual smears were performed and stained using Pappenheim's method (May-Grünwald-Giemsa (MGG) staining: May-Grünwald dye for 3–5 minutes and then Giemsa dye for 13 to 15 minutes). The drained, stained smears were viewed under a light microscope (100x magnification). The percentage composition of peripheral blood leukocytes was estimated. The percentage of leukocytes was evaluated.

Inflammatory markers were also evaluated at each step of EPT.

### 2.8. Statistical Analysis

To evaluate the differences between the independent groups (not related to each other), Mann-Whitney *U* test was used. In the case of dependent groups, Wilcoxon's matched pairs test was used. To compare more than two groups, the Kruskal-Wallis ANOVA test was used. In all tests, the statistically significant level was *α* < 0.05. Results were presented as mean IKB ± SD.

## 3. Results

**Figure 1:**
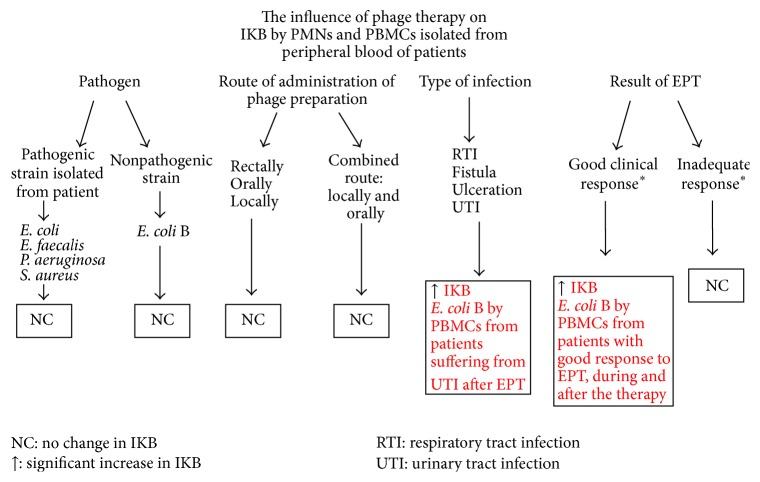
Analysis of the influence of phage therapy on the ability of phagocytes (PMNs and PBMCs) isolated from peripheral blood of patients to kill bacteria intracellularly.  ^*∗*^Effectiveness evaluation of EPT according to Międzybrodzki et al. (2012) [14].

In our study, phagocytes isolated from peripheral blood, PMNs and PBMCs (containing monocytes), of each patient were tested and the ability of these cells to intracellularly kill the pathogenic bacterial strain (antibiotic-resistant strain isolated from the patient, which was the cause of the infection) and the nonpathogenic one (*E. coli* B) was checked (Table 1). The scheme of the analysis of the influence of phage therapy on the ability of phagocytes (PMNs and PBMCs) isolated from patients' blood to kill bacteria intracellularly is presented in Figure 1.

We observed that both PMNs and PBMCs from patients with infection caused by* E. coli* were characterized by weakened ability to kill bacteria intracellularly before initiating the therapy with the phages in these patients (Mann-Whitney *U* test, *p* = 0.001 and *p* = 0.003). The bactericidal ability of PMNs from patients to kill* E. coli* strains intracellularly during the therapy was reduced (a statistically significant difference was observed between healthy donors and patients' PMNs' ability to kill bacteria intracellularly during EPT, *p* = 0.018). However, the treatment itself did not cause further reduction in IKB. Patients' PMNs after EPT showed no weakness in IKB (*p* = 0.168). It was not observed that IKB by PBMCs was weakened during and after EPT (statistically significant differences were not observed between the ability to kill* E. coli* strains shown by healthy volunteers and patients' phagocytes during and after phage treatment; Mann-Whitney *U* test, *p* = 0.056 and *p* = 0.082, resp.). These results indicate that the use of phage lysates active against* E. coli* did not cause a further decrease in the bactericidal capacity of patients' peripheral blood phagocytes.

Both PMNs and PBMCs isolated from patients with infection caused by* E. faecalis* showed a reduced ability to kill bacteria intracellularly before treatment with the phages in the studied group of patients (Mann-Whitney *U* test indicated that the observed differences are statistically significant, *p* = 0.004). The bactericidal capacity of patients' PMNs was decreased both during and after the use of phage lysates containing phages active against* E. faecalis* (statistically significant differences in the ability of neutrophils of both healthy individuals and patients to kill bacteria intracellularly during therapy, *p* = 0.001, and after the treatment, *p* = 0.004), but the treatment did not further reduce it. IKB by PBMCs before the therapy was weakened (Mann-Whitney *U* test indicated that the difference in bactericidal ability of PBMCs in the group of patients in which* E. faecalis* constituted the cause of infection, when compared to healthy donors, is significant, *p* = 0.001). Killing of* E. faecalis* strain by patients' PBMCs during EPT was decreased (significant difference in the killing activity of* E. faecalis* between patients' and healthy PBMCs, *p* = 0.001). After the therapy the intracellular killing of* E. faecalis* by patients' mononuclear cells was not decreased (no statistically significant differences were detected in the ability to kill* E. faecalis* shown by PBMCs of healthy individuals and patients after application of phages; Mann-Whitney *U* test, *p* = 0.062).

Neutrophils and monocytes of patients with infections caused by* P. aeruginosa* showed a decreased ability to kill bacteria intracellularly prior to EPT, during phage application, and after the treatment (significant differences in the killing activity of* P. aeruginosa* between patients' and healthy PMNs as well as PBMCs were observed; Mann-Whitney *U* test, *p* = 0.001).

As a result of our experiments we observed that both neutrophils and monocytes of patients with infections caused by* S. aureus* showed a reduced ability to kill bacteria intracellularly prior to, during, and after EPT. These observations indicate that phage therapy does not affect the ability of phagocytes to kill bacteria intracellularly in patients in whom the cause of infection was* S. aureus*.

Apart from the tested phagocytic ability of the patient to kill the pathogenic bacteria, the phagocytic ability to kill nonpathogenic* E. coli* B strain was examined also. While comparing the ability of both patient's PMNs and PBMCs to kill* E. coli* B at all stages of phage therapy, whether before, during, or after the application of phage preparations, to the ability of phagocytes isolated from healthy volunteers, significant differences were observed (Mann-Whitney *U* test, *p* = 0.001).

It was observed that intracellular killing of pathogenic strains,* E. coli*,* E. faecalis, P. aeruginosa*, and* S. aureus*, isolated from patients treated with phages and the reference (nonpathogenic)* E. coli* B strain was significantly reduced before EPT compared to the bactericidal activity of phagocytes (PBMCs and PMNs) from healthy individuals. Furthermore, the reduced ability to kill bacteria intracellularly by both PMNs and PBMCs was observed during and after the treatment (as compared to the control group) with the exception of pathogenic strains of* E. coli* and* E. faecalis*. The results indicate that the use of phage lysates did not exacerbate the defect in IKB observed in the case of peripheral blood phagocytes from patients qualified for EPT and treated with phages.

Chronic bacterial infections in patients cause IKB impairment. The administration of phage lysates did not further decrease the observed deficit. The intracellular killing of pathogenic strains,* E. coli*,* E. faecalis, P. aeruginosa*, and* S. aureus*, of patients' blood phagocytes prior to EPT was significantly weaker (*p* < 0.005). We also observed significantly weaker intracellular killing of the reference* E. coli* B strain by patients' PMNs and monocytes when compared to healthy controls. There were no changes in the ability to kill pathogenic strains at subsequent stages of phage treatment.

It was also tested whether IKB is different for the pathogenic strain at each stage of phage therapy and whether there are differences in IKB between groups of patients from whom the same species of bacteria were isolated, as well as whether the route of phage administration and the type of infection have any impact on the ability of phagocytes (isolated from peripheral blood of patients) to kill bacteria intracellularly.

An analysis of intracellular killing of pathogenic strains (*P. aeruginosa*,* E. coli*,* E. faecalis*, and* S. aureus*) isolated from patients, between the groups of patients treated with preparations applied through different routes, rectally, orally, topically, or topically and orally, was performed using the Kruskal-Wallis test (nonparametric ANOVA). The results are presented in Table 2. There were no significant differences between the groups (*p* > 0.05) at different stages of phage therapy (before and during the application of the lysate and after the treatment); therefore, it can be assumed that the route of administration of phage lysate does not significantly affect IKB by peripheral blood phagocytes, both PMNs and PBMCs, and does not vary at different stages of treatment in the studied groups of patients. The type of infection has no significant effect on the tested IKB by peripheral blood phagocytes and also does not vary at different stages of treatment in the study group (Table 4).

**Figure 2:**
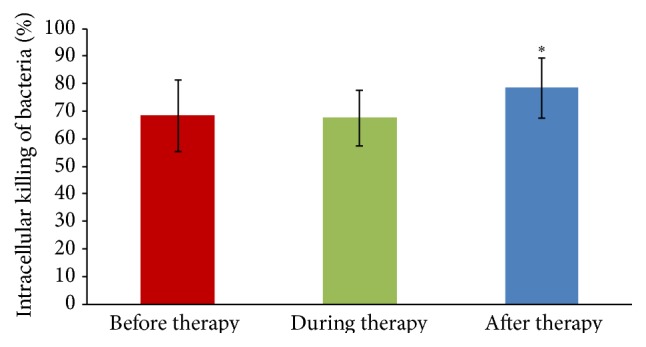
Mean intracellular killing of* E. coli* B (±SD) by monocytes (in the mononuclear cell suspension) isolated from patients with urinary tract infections before (*n* = 18), during (*n* = 17), and after phage therapy (*n* = 13).  ^*∗*^Statistically significant increase of IKB observed after EPT compared to the value at the beginning of EPT (Mann-Whitney *U* test).

We compared the patients' blood phagocytes' ability to kill intracellularly the nonpathogenic strain* E. coli* B between groups of patients with different types of infections. Only in the case of intracellular killing of* E. coli* B by PBMCs isolated from peripheral blood of patients suffering from urinary tract infection (UTI) was a significant increase of IKB observed after the phage treatment (78.5% ± 11.0) compared to the value at the beginning of phage treatment (68.4% ± 13.1), *p* = 0.02, as shown in Figure 2.

**Figure 3:**
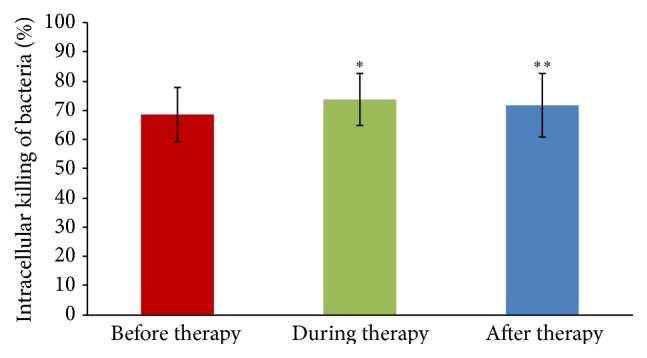
Mean intracellular killing of* E. coli* B (±SD) by PBMCs isolated from patients with good response to phage therapy before (*n* = 19), during (*n* = 17), and after phage therapy (*n* = 13).  ^*∗*^Statistically significant increase of IKB observed during EPT compared to the value at the beginning of EPT (Mann-Whitney *U* test).  ^*∗∗*^Statistically significant increase of IKB observed after EPT compared to the value at the beginning of phage treatment.

We studied also the differences in IKB of patients with good and inadequate response to EPT. A statistically significant increase in patients' monocytes' killing ability during (73.6% ± 8.8) and after the therapy (71.8% ± 11.0) was observed in the case of* E. coli* B strain in patients with a good response to EPT (37.3% of patients from the tested group) compared to IKB before EPT (68.5% ± 9.3), *p* = 0.04 and *p* = 0.02, respectively, as presented in Figure 3. No differences were observed in the course of PT in these patients for IKB of pathogenic bacteria or in IKB by PMNs. No significant changes in IKB of patients inadequately responding to EPT were observed.

**Figure 4:**
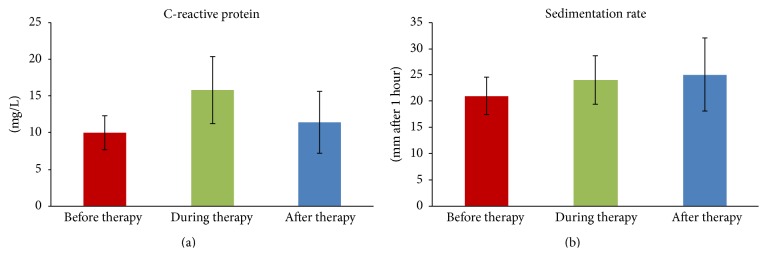
Mean level of inflammatory markers (±SD). (a) CRP. (b) Sedimentation rate in patients' peripheral blood before (*n* = 39), during (*n* = 28), and after (*n* = 15) EPT.

**Figure 5:**
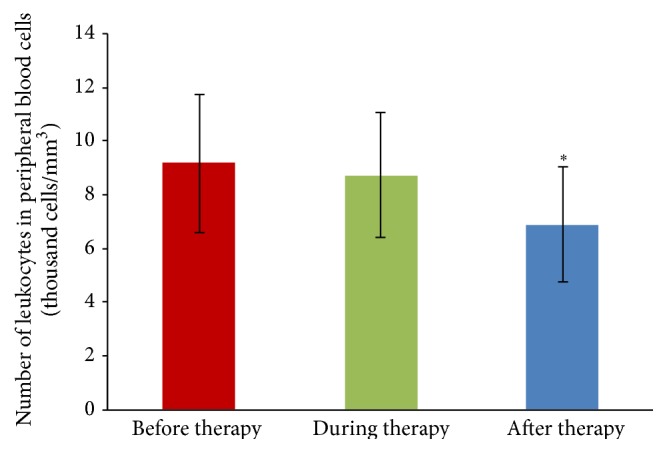
Leukocytosis in patients with infections caused by* P. aeruginosa* treated with EPT before (*n* = 10), during (*n* = 8), and after the treatment (*n* = 4). The differences were tested using Wilcoxon's test.  ^*∗*^Statistically significant difference between the number of leukocytes in the peripheral blood of patients after EPT and the number of leukocytes before the treatment, Wilcoxon's test.

It was observed that EPT has no effect on the number of leukocytes and lymphocytes circulating in peripheral blood (Table 3) or on the level of inflammatory markers, ESR and CRP (Figure 4) determined at each step of the phage preparation application (*p* > 0.05) and compared with control (blood from healthy volunteers), because we did not observe any significant changes in the percentage of different types of leukocytes or inflammatory markers (Wilcoxon test, *p* > 0.05). However, it was shown that EPT causes only a significant decrease in the number of leukocytes circulating in the peripheral blood after the termination of treatment (6.9 ± 2.3 thousand cells/mm^3^ of blood) compared to values before the phage therapy (9.2 ± 4.2 thousand cells/mm^3^ of blood) in patients infected with* P. aeruginosa*, presented in Figure 5.

## 4. Discussion

The studies presented in this paper concern the influence of phage preparations (applied in EPT) on bactericidal activity of phagocytes responsible for engulfing and destroying antigens inside phagosomes in the IKB process (which constitutes the fundamental mechanism of immune defense against pathogens).

The course of bacterial infection and the effectiveness of antimicrobial therapy depend not only on the sensitivity of the pathogen to the applied drug but also on many other factors, such as the concentration/level of the applied antimicrobial substance, the reactivity of the immune system, and the possible interaction of the applied agent with elements of the immune system, including phagocytes [9, 21, 22]. The effect of phage preparations on the immune system depends on several factors such as the type of phage, the type of preparation, its form, route of administration, length of therapy, and whether phage neutralizing antibodies are present in the blood [17].

Some diseases, for example, innate immunodeficiencies (e.g., chronic granulomatous disease), and chronic or recurrent bacterial infections may cause the bactericidal activity of phagocytes to be impaired. Acute bacterial infections can also cause temporary impairment of phagocytic bactericidal function [23]. Bacterial infections, for example, acute UTI in the mouse model, cause IKB impairment (Jończyk-Matysiak et al., unpublished data). Some drugs (e.g., phenylbutazone, metamizole, and suramin) can also cause a similar effect [24]. Antibiotic therapy can lead to the release of components of the cell wall of bacteria, sometimes resulting in complications in the course of a bacterial infection, for example, sepsis or septic shock [25, 26]. Moreover, bacterial cell wall components (e.g., M protein produced by* S. pyogenes* and mycolic acid produced by* M. tuberculosis*) and metabolic products of bacteria (e.g., succinic acid or catalase produced by* L. monocytogenes*) can also contribute to the impairment of phagocytosis or IKB [27, 28] increasing the likelihood of pathogen survival and development of the infection. Disorders of the bactericidal activity of phagocytes of both PMNs and PBMCs in the group of patients whom we studied could be due to innate or acquired immunodeficiency (causing impairment of the IKB process) which contributes to the occurrence or increase in the probability of infection, both chronic and recurrent [29]. Weakened immunity could be a consequence of the use of antibiotics [30] or of immunosuppressive drugs (e.g., in the case of kidney transplant patients: patients have impaired bacterial killing by neutrophils) [31].

According to the Phage Therapy Unit's data, approximately 50% of patients treated in the Phage Therapy Unit had their immunity impaired [14, 17]. Peripheral blood phagocytes from the tested group of patients were initially characterized by weakened ability to kill intracellularly both the bacterial pathogenic strain that was the cause of infection and nonpathogenic (reference strain)* E. coli* B. Moreover, the impairment of bactericidal activity of these phagocytes was maintained during and after the therapy (despite the increasing trend observed for IKB by patients' phagocytes). The obtained results showed only a significant improvement in intracellular killing of nonpathogenic* E. coli* B strain by patients' monocytes. This could be due to differences in the structure, the presence of different surface structures (e.g., polysaccharide capsule), and the expression of cell surface receptors that distinguish pathogenic from nonpathogenic bacteria. In particular, the structure and properties of the bacterial cell surface are important for the interaction of microbe-phagocyte layout [32]. The presence of crystalline surface protein (e.g., in* Bacillus cereus*) forming the S-layer (responsible for bacterial adhesion and interactions between host and bacterial cells) on* B. cereus* surface protected reference strains against phagocytosis, whereas the lack of this layer on clinical isolates made them susceptible to phagocytosis. According to Kotiranta et al., 1998, both reference strains and 1-day cultures of clinical strains were characterized by hydrophilicity, whereas 3-day and 6-day cultures of pathogenic strains were hydrophobic. This characteristic appeared with the “age” of bacterial culture. One-day clinical strains were phagocytosed by human neutrophils during 30 min. The reference strains and 3-day or 6-day pathogenic strains were characterized by resistance to phagocytosis, and this phenomenon was explained as loss of sites/receptors binding neutrophils in those bacteria. According to van Oss et al., 1983, nonpathogenic strains were hydrophilic and were easily phagocytosed [33]. But pathogenic bacteria were resistant to engulfment by phagocytes, which was the result of the presence of the hydrophilic outer surface.

Differences in alveolar macrophage phenotype were observed after 1 day of* Haemophilus parasuis* infection in animals (pigs) infected with a nonpathogenic and a pathogenic bacterial strain [34]. This nonpathogenic strain induced higher levels of CD163 on the surface of macrophages, reflecting the strain's susceptibility to phagocytosis and IKB by macrophages. The reduced expression of CD172a, CD163, and Swine II histocompatibility leukocyte antigen SLA II resulted in the impossibility of macrophage activation by the pathogenic strain. It is suggested that an early response to a pathogenic strain is necessary for its removal, while the initial inhibition of the inflammatory response by the pathogenic strain could lead to the spread of infection. Differences in the interactions of pathogenic and nonpathogenic strains with human neutrophils have also been observed in the case of* Streptococcus pyogenes* strains [35]. The bacteria expressing the M or M-like protein avoid being killed by neutrophils by preventing the fusion of azurophilic granules with phagosomes, whereas nonpathogenic mutants were killed and degraded in the phagosome, with which the azurophilic granules were fused.

The defective intracellular killing of* Coxiella burnetii* by monocytes characterized patients suffering from endocarditis (chronic Q fever) caused by* C. burnetii* [36]. This defect in intracellular killing of* C. burnetii* affected early and delayed steps of monocyte bactericidal activity. Interestingly, the addition of supernatants of* C. burnetii*-stimulated monocytes from patients with active endocarditis to control monocytes (from healthy subjects) supported* C. burnetii* survival. These observations suggested that some factor was responsible for bacterial survival, and the authors observed that tumor necrosis factor (TNF) was involved in this defect. Monocytes of patients with endocarditis, which were not able to eliminate* C. burnetii*, secreted high levels of TNF in response to* C. burnetii*. What is more, monocytes of patients with chronic Q fever showed impaired* C. burnetii* killing because of defective phagosome maturation [37].

We did not study in detail phage effects on particular interactions between bacteria and phagocytes. We had been interested in the final effect of phage treatment on IKB. As a result of our investigations we found that both pathogenic and nonpathogenic bacterial strains are phagocytosed and killed inside phagocytes. The EPT does not adjust the reduced ability of PMNs and PBMCs to intracellular killing of the pathogenic strain, which is the cause of infections in the studied patients. We observed only a significant increase in PBMCs' ability to kill the nonpathogenic* E. coli* B strain, whereas in the case of PMNs only an upward trend was noted.

During and after the EPT, a significant increase in monocytes' ability to kill the nonpathogenic control strain was observed (in the case of a good response to therapy). The bactericidal activity of monocytes could be impaired/lowered by the impact that the pathogenic strain had on them (in the case of chronic infections), probably because we did not observe any increase in bactericidal activity towards this type of bacterial strains. This could manifest with the suppression of respiratory burst or slower fusion of the phagosome membrane with the lysosome. Interestingly, according to Levine et al., 2014, the proper functioning of neutrophils is connected with the HVCN1 proton channel, which is involved in sustainable proton transport across the vacuolar membrane [38]. The impairment of its activity may be due to the excessive acidification of cytosol, which may be the cause of inhibited NADPH oxidase activity. Perhaps in patients with reduced ability of neutrophils to kill bacteria intracellularly, the weakness could be due to a failure of the HVCN1 channel, and therefore a significant increase in IKB was observed only in the case of monocytes of patients.

Phage preparations used in the treatment of the patients were in the form of lysates, which contain phage virions suspended in LB medium or peptone water. Therefore, in their composition, there were products of the metabolism or degradation of the bacteria and culture media components. Accordingly, the content of these components could cause the interaction of phagocytes to depend not only on the interaction of phage particles with PMNs and PBMCs but also on the level of bacterial remains (e.g., endotoxins).

The literature data suggest that a major barrier in the interpretation of the results of the testing of phagocytes* in vitro* may result from unspecific activation of phagocytes, which can be observed during the isolation and preparation of cells [39, 40]. Neutrophils isolated from blood may have different characteristics as compared to those that reach the tissues. Therefore we used the method of cell isolation, involving density gradient centrifugation, which is now commonly used in such studies [40]. We decided to test the activity of monocytes in a suspension of whole fraction of peripheral blood mononuclear cells because the isolation of pure monocytes would require collecting much larger samples of blood [39] that could not be ethically acceptable in our group of chronically sick patients with an ongoing disease process who often suffer from anemia. However, in a fraction of mononuclear cells, monocytes are the only cells with phagocytic ability, because lymphocytes do not show phagocytic properties. In order to exclude immunodeficiencies, the percentage of isolated monocytes in the fraction of PBMC was evaluated. The IKB was calculated taking into account the fraction of cells containing monocytes both in the suspension in which only phagocytosis occurred and in the suspension in which phagocytosis and IKB occurred. We did not observe statistically significant differences in the percentage of monocytes in PBMCs between groups of patients before EPT (14.6% ± 4.1), during therapy (13.9% ± 3.8), and after the therapy (14.7% ± 5.4) (Wilcoxon's test, *p* > 0.05) (not presented herein, Jończyk-Matysiak et al., unpublished data). The obtained data showed that slight fluctuations in the percentage of monocytes may not be responsible for changes in IKB by PBMCs.

Our results confirm the observations made by Kurzepa-Skaradzinska et al., 2013,* in vitro* [41]. They demonstrated that phage preparations, irrespective of their form (whether in the lysate or purified preparation form), the titer, and specificity or the bacterial strain used (homologous and heterologous) did not affect the ability of human phagocytes (from healthy donors) to kill bacteria intracellularly. The investigations of the effect of phage therapy on phagocytosis of* S. aureus* by neutrophils of patients subjected to experimental phage therapy (EPT) showed a further decrease in the bactericidal activity in patients whose phagocytes were characterized by decreased activity before the therapy [42]. In addition, there was no correlation between changes in phagocytosis and the course of treatment.

Interestingly, the synergistic action of PA1Ø phage (active against* P. aeruginosa*) and human neutrophils isolated from peripheral blood was observed against* P. aeruginosa in vitro* [43]. There was a significant decrease in bacterial titer in the samples which contained phage and neutrophils when compared to samples containing the phage only. Our unpublished studies (Jończyk-Matysiak et al.) showed that preincubation of the purified T4 phage with PMNs or PBMCs significantly increases the percentage of killed bacteria, compared to bacteria incubated with the same phagocytes. These data indicate that the interaction of phages and phagocytes in the elimination of bacteria does occur. Moreover, it was observed that the T4 phage can penetrate into the interior of murine macrophages [44]. Kaur et al. (2014) demonstrated that MR-5 phage specific to* S. aureus* (adsorbed to bacterial cells) penetrated murine macrophages and caused a significant reduction in* S. aureus* titer (a decrease of 2.5 log during 2 h) that was localized intracellularly [45]. The phage particles, which were transferred to the interior of macrophages, significantly reduced the damage due to cytotoxic effects of bacteria on phagocytes.

Patients were qualified for phage therapy after prior ineffective antibiotic treatment; thus weakening the bactericidal function of phagocytes could also result from phenotypic features of strains. Antibiotic-resistant strains such as MRSA may be characterized by weakened IKB by neutrophils [46]. The growing antibiotic resistance of bacterial strains [47, 48] causes bacteriophages to be considered a potential alternative to antibiotics. Wenisch et al., 1996, found that the use of a single oral dose of azithromycin decreased* E. coli* phagocytosis by neutrophils of healthy individuals by 38% (compared to phagocytosis before antibiotic application) as well as the production of free radicals (a decrease of 25%) [49].

The use of antibiotics is a factor that induces antibiotic resistance (it increases in successive cycles of treatment) in the bacteria. Moreover, antibiotics destroy both pathogenic strains and symbiotic ones (i.e., producing stimulant substances, e.g., in the gut immune system) [50], which may impair intestinal innate immune mechanisms in both humans and mice [51, 52]. Antibiotics can also cause allergic reactions [53]. There is also evidence that some of these drugs, for example, chloramphenicol, may have toxic effects on bone marrow, resulting in aplastic anemia [54]. The available data suggest that phage therapy is effective in the treatment of infections caused by antibiotic-resistant strains [14, 55–62]. Moreover, the use of bacteriophages can be safe in patients with immunodeficiency [63].

Phage lysates can be used as active agents modulating the immune system. The observed strengthening of immunity may be caused by the products contained in the preparations, for example,* staphylococcal* phage lysates [17]. As mentioned by Górski et al., 2012, phage preparations' therapeutic effect not only may be related to the elimination of bacteria but also may depend on the normalization of inflammatory markers associated with bacterial infection. Recent reports of Stapels et al., 2014, suggest that* S. aureus* has the ability to produce serine protease inhibitors, which could be important in the inhibition of the inflammatory process [64]. This finding would suggest a potential mechanism for the anti-inflammatory effects of phage lysates active against staphylococci.

*In vitro* experiments showed that the purified T4 phage induced respiratory burst in monocytes and neutrophils much weaker than bacteria [13]. It also inhibits the production of ROS by neutrophils stimulated with bacteria and lipopolysaccharide (LPS). Similarly, neither the purified staphylococcal A3 phage nor A3 lysate induced respiratory burst in either neutrophils or monocytes [65]. Interestingly, Gorczyca et al. (2007) observed that addition of T4 and A5 phage to a culture of mononuclear cells infected with human herpesvirus-1 (HHV-1) caused inhibition of production of nuclear factor *κ*B (NF*κ*B), a transcription factor responsible for the expression of many genes (including genes encoding proinflammatory cytokines) [66].

The influence of T4 and A3R lysates and purified preparations on differentiation of dendritic cells from human peripheral blood monocytes* in vitro* was examined. Korczak-Kowalska (unpublished data) showed that the purified preparations did not induce the expression of CD40, CD80, CD83, CD86, CD1c, CD11c, MHC II, PD-L1, PD-L2, TLR2, TLR4, and CCR7 molecules and receptors of phagocytosis CD64 and DEC-205. The purified phage preparations did not cause the expression of these molecules. Moreover, T4 phage lysate decreased the percentage of dendritic cells with the expression of CD1c and DEC-205, whereas A3R lysate also decreased the expression of DEC-205 and both tested lysates inhibited the differentiation of dendritic cells.

There is evidence that some antibiotics, for example, chloramphenicol, may have toxic effects on bone marrow, resulting in, for example, aplastic anemia, or they may induce neutropenia, which may lead to immunity impairment [29, 30]. Previous research conducted in our laboratory demonstrated that phage therapy accelerates the circulation of patients' neutrophils, which was confirmed by a significant increase in the number of immature forms of neutrophils (with a band-shaped nucleus) in the peripheral blood, with a simultaneous decrease in mature cells (with a segmented nucleus) [42]. At least in some patients (treated with phage therapy) a decrease of serum C-reactive protein (CRP) could also be observed [67]. The results presented herein suggest that EPT has no effect on the number of leukocytes and lymphocytes circulating in peripheral blood or on the level of the inflammatory markers ESR and CRP, which may confirm our earlier results revealing the impact of phage therapy on the functions of human organs [14].

## 5. Conclusions

Our original results contribute to the understanding of the interaction between phages and phagocytes and this paper is the first presenting the effect of bacteriophage preparations on intracellular killing of bacteria by phagocytes in patients. We have shown that chronic bacterial infections (caused by antibiotic-resistant bacterial strains) in patients qualified for EPT caused the impairment of peripheral blood phagocytes' (both PMNs and PBMCs) ability to kill bacteria intracellularly, both pathogenic and nonpathogenic ones. Moreover, we demonstrated that phage therapy did not decrease patients' phagocytes' ability to kill bacteria (both the pathogenic and the reference strain). In particular, in patients with initially reduced ability to kill bacteria intracellularly, we did not observe a further decrease in IKB by phagocytes. What is more, phage treatment may correct the weakened IKB by monocytes in the case of the nonpathogenic strain. Our results confirm that phage therapy has no harmful effects on the bactericidal properties of peripheral blood phagocytes isolated from patients treated with phage lysates. Moreover, EPT does not appear to have a significant impact on the percentage of the different fractions of peripheral blood leukocytes, so it probably does not affect the granulopoiesis or myelopoiesis. There was no significant influence of phage lysates on inflammatory markers in patients who received phage therapy, which may suggest that EPT does not stimulate the inflammatory process. The obtained results may have practical implications as they confirm safe usage of phage preparations in patients treated with phage therapy, especially in patients with primary and secondary immunodeficiencies.

## Figures and Tables

**Table 1:** Intracellular killing of nonpathogenic and pathogenic strains compared to control.

Bacteria	Tested group of patients	*n*	Mean IKB by PMNs ± [%]	*n*	Mean IKB by PBMCs ± [%]
*E. coli B*	Control	39	87.0 ± 8.1	39	86.8 ± 8.8
	Before therapy	51	69.0 ± 13.0^*∗*^	51	69.2 ± 10.6^*∗*^
	During therapy	44	71.0 ± 10.2^*∗*^	44	71.4 ± 10.6^*∗*^
	After therapy	33	71.0 ± 12.9^*∗*^	33	76.0 ± 12.4^*∗*^

*E. coli *	Control	23	85.8 ± 7.5	23	85.7 ± 7.7
	Before therapy	7	72.6 ± 8.1^*∗*^	7	71.0 ± 12.2^*∗*^
	During therapy	6	75.4 ± 9.1^*∗*^	6	75.9 ± 12.7
	After therapy	5	75.8 ± 15.3	5	72.5 ± 17.3

*E. faecalis*	Control	25	88.0 ± 7.8	25	84.4 ± 7.2
	Before therapy	7	73.0 ± 11.5^*∗*^	7	73.2 ± 8.5^*∗*^
	During therapy	7	65.0 ± 16.5^*∗*^	7	66.8 ± 15.3^*∗*^
	After therapy	4	72.0 ± 5.6^*∗*^	4	75.9 ± 8.6

*P. aeruginosa*	Control	25	88.0 ± 6.5	25	87.9 ± 7.5
	Before therapy	11	71.5 ± 11.4^*∗*^	11	67.8 ± 13.7^*∗*^
	During therapy	10	72.4 ± 6.3^*∗*^	10	73.1 ± 8.7^*∗*^
	After therapy	7	69.5 ± 7.7^*∗*^	7	70.6 ± 6.1^*∗*^

*S. aureus*	Control	25	88.0 ± 6.7	25	88.3 ± 7.4
	Before therapy	23	74.0 ± 8.6^*∗*^	23	71.9 ± 8.5^*∗*^
	During therapy	18	70.10 ± 10.2^*∗*^	18	71.7 ± 11.4^*∗*^
	After therapy	14	70.80 ± 11.6^*∗*^	14	74.2 ± 9.6^*∗*^

^*∗*^The statistically significant difference between tested group and control (Mann-Whitney *U* test).

**Table 2:** Intracellular killing of pathogenic strains depending on the route of administration of phage preparations.

Route of phage administration	IKB by PMNs before EPT [%]	*n*	IKB by PMNs during EPT [%]	*n*	IKB by PMNs after EPT [%]	*n*	IKB by PBMCs before EPT [%]	*n*	IKB by PBMCs during EPT [%]	*n*	IKB by PBMCs after EPT [%]	*n*
Orally	65.4 ± 10.4	5	71.1 ± 8.2	4	67.2 ± 10.8	3	66.2 ± 20.0	5	71.2 ± 7.5	4	69.0 ± 4.3	3
Rectally	73.5 ± 9.0	12	64.8 ± 13.5	12	69.9 ± 7.3	10	71.4 ± 10.8	12	69.2 ± 12.7	12	72.5 ± 10.7	10
Locally	74.2 ± 9.6	27	72.1 ± 7.8	21	71.1 ± 13.9	18	72.4 ± 9.2	27	70.8 ± 11.7	21	75.5 ± 9.8	18
Locally and orally	69.6 ± 8.5	7	72.8 ± 10.1	7	78.0 ± 13.4	2	74.8 ± 5.9	7	78.8 ± 9.2	7	78.0 ± 9.2	2

**Table 3:** Peripheral blood smear before, during, and/or after EPT.

Mean ± SD	Granulocytes with band- shaped nucleus [%]	*n*	Granulocytes with segmented nucleus [%]	*n*	Eosinophils [%]	*n*	Basophils [%]	*n*	Lymphocytes [%]	*n*	Monocytes [%]	*n*
Control	3.9 ± 1.3	12	53.8 ± 7.7	12	2.75 ± 1.5	12	0.5 ± 0.8	12	32.1 ± 6.6	12	7.0 ± 2.3	12
Before therapy	3.8 ± 2.0	22	51.1 ± 10.5	22	4.3 ± 9.5	22	0.3 ± 0.4	22	35.6 ± 11.3	22	7.1 ± 2.3	22
During therapy	3.0 ± 0.9	22	49.2 ± 15.7	22	2.9 ± 3.2	23	0.3 ± 0.5	22	36.2 ± 12.3	23	6.6 ± 2.2	22
After therapy	3.2 ± 1.3	10	51.1 ± 11.6	10	3.3 ± 2.5	10	0.1 ± 0.3	10	35.3 ± 12.7	10	7.1 ± 2.0	10

**Table 4:** Mean intracellular killing of pathogenic strains according to the type of infection (mean ± SD).

Type of infection	IKB by PMN before EPT [%]	*n*	IKB by PMN during EPT [%]	*n*	IKB by PMN after EPT [%]	*n*	IKB by PBMC before EPT [%]	*n*	IKB by PBMC during EPT [%]	*n*	IKB by PBMC after EPT [%]	*n*
*E. coli*												
UTI^*∗*^	72.6 ± 8.1	7	75.4 ± 9.1	6	75.8 ± 15.3	5	71.0 ± 12.2	7	75.9 ± 12.7	6	72.5 ± 17.3	5

*E. faecalis*												
UTI	72.7 ± 11.5	7	65.0 ± 16.5	7	72.1 ± 5.6	4	73.2 ± 8.5	7	66.8 ± 15.3	7	75.9 ± 8.6	4

*P. aeruginosa*												
RTI^*∗∗*^	64.4 ± 11.2	4	67.9 ± 7.9	3	72.6 ± 7.6	2	66.6 ± 13.6	4	73.3 ± 5.1	3	66.8 ± 2.5	2
Fistula	81.0 ± 0.0	1	75.0 ± 0.0	1	68.0 ± 0.0	1	79.5 ± 0.0	1	82.0 ± 0.0	1	82.0 ± 0.0	1
UTI	72.3 ± 0.4	2	74.8 ± 3.2	2	66.4 ± 14.0	2	61.8 ± 25.6	2	68.9 ± 8.7	2	70.3 ± 4.6	2
Ulceration	75.9 ± 13.7	4	73.9 ± 6.8	4	70.3 ± 7.4	2	69.0 ± 11.3	4	72.9 ± 12.2	4	69.3 ± 6.0	2
Mean for all types	**71.5 ± 11.4**	**11**	**72.4 ± 6.3**	**10**	**69.5 ± 7.7**	**7**	**67.8 ± 13.7**	**11**	**73.1 ± 8.7**	**10**	**70.6 ± 6.1**	**7**

*S. aureus*												
RTI	74.4 ± 9.7	4	70.0 ± 12.1	4	80.3 ± 16.6	2	72.4 ± 5.3	4	73.7 ± 8.9	4	78.3 ± 9.5	2
Fistula	74.7 ± 9.1	11	73.9 ± 8.0	7	62.9 ± 16.0	5	77.0 ± 8.8	11	75.8 ± 12.5	7	74.3 ± 7.3	5
UTI		0		0		0		0		0		0
Ulceration	72.8 ± 8.5	8	68.0 ± 7.2	7	71.4 ± 11.4	7	73.0 ± 8.9	8	66.4 ± 12.4	7	75.0 ± 10.4	7
Mean for all types	**74.0 ± 8.6**	**23**	**70.8 ± 8.6**	**18**	**69.6 ± 14.0**	**14**	**74.8 ± 8.3**	**23**	**71.7 ± 11.9**	**18**	**75.2 ± 8.7**	**14**

^*∗*^UTI: urinary tract infection.

^*∗∗*^RTI: respiratory tract infection.

## Abbreviations

EPT: Experimental phage therapy
FCS: Fetal calf serum
IIET PAS: Institute of Immunology and Experimental Therapy of the Polish Academy of Sciences
IKB: Intracellular killing of bacteria
LPS: Lipopolysaccharide
PBMCs: Peripheral blood mononuclear cells
PBS: Phosphate-buffered saline
PMNs: Polymorphonuclear neutrophils
ROS: Reactive oxygen species.
